# Mycolactone cytotoxicity in Schwann cells could explain nerve damage in Buruli ulcer

**DOI:** 10.1371/journal.pntd.0005834

**Published:** 2017-08-04

**Authors:** Junichiro En, Sho Kitamoto, Akira Kawashima, Suguru Yonezawa, Yoshito Kishi, Norihisa Ishii, Masamichi Goto

**Affiliations:** 1 Department of Pathology, Kagoshima University, Kagoshima, Japan; 2 National Sanatorium Hoshizuka-Keiaien, Kanoya, Kagoshima, Japan; 3 International University of Health and Welfare, Narita, Chiba, Japan; 4 Division of Gastroenterology, Department of Internal Medicine, University of Michigan Medical School, Ann Arbor, Michigan, United States of America; 5 Department of Clinical Laboratory Science, Faculty of Medical Technology, Teikyo University, Itabashi, Tokyo, Japan; 6 Department of Chemistry and Chemical Biology, Harvard University, Boston, Massachusetts, United States of America; 7 Leprosy Research Center, National Institute of Infectious Diseases, Higashimurayama, Tokyo, Japan; Kwame Nkrumah University of Science and Technology, GHANA

## Abstract

Buruli ulcer is a chronic painless skin disease caused by *Mycobacterium ulcerans*. The local nerve damage induced by *M*. *ulcerans* invasion is similar to the nerve damage evoked by the injection of mycolactone in a Buruli ulcer mouse model. In order to elucidate the mechanism of this nerve damage, we tested and compared the cytotoxic effect of synthetic mycolactone A/B on cultured Schwann cells, fibroblasts and macrophages. Mycolactone induced much higher cell death and apoptosis in Schwann cell line SW10 than in fibroblast line L929. These results suggest that mycolactone is a key substance in the production of nerve damage of Buruli ulcer.

## Author summary

Buruli ulcer is a chronic skin disease caused by *Mycobacterium ulcerans*, and the disease is characterized by the painless nature of its lesion. Similar to leprosy, loss of pain often hinders the patients from taking proper medical care, resulting in gross deformities. A toxic lipid mycolactone produced from *Mycobacterium ulcerans* was thought to block the sensory system of the lesion, either by direct cellular damage (cytotoxicity) to the regional nerve tissue, or by a more sophisticated, non-toxic paralyzing mechanism. In the peripheral nerve, Schwann cells nourish axons and accelerate nerve conduction. In this study, we have compared the cytotoxic potential of mycolactone on cultured Schwann cells and that on fibroblasts, and found that mycolactone A/B induced much higher cell death and apoptosis in Schwann cell line SW10 than in fibroblast line L929. These results support the cytotoxic theory and suggest that mycolactone is a key substance in the production of nerve damage of Buruli ulcer.

## Introduction

Buruli ulcer is a disease characterized by the painless nature of its lesion. The disease is basically characterized by the ulcer without pain [1], but some pain is noted at the wound care dressing service [2]. These studies suggest that Buruli ulcer lesions are initially painless, but the patients experience pain after chemotherapy, probably due to nerve regeneration. Studies of the pathological mechanism have revealed that local nerves are invaded and damaged by the causative agent, *M*. *ulcerans* [3], and that similar nerve damage is evoked by the injection of mycolactone in a mouse model [4]. In both instances, Schwann cells, which play the major role in maintaining nerve function, showed vacuolar degeneration. Also, nerve damage was histopathologically confirmed in human Buruli ulcer lesions [5].

To further elucidate the mechanism of nerve damage in Buruli ulcer, we tested the cytotoxic effect of mycolactone on a cultured Schwann cell line (SW10). Because mycolactone evokes cell death and apoptosis in fibroblasts [6, 7], macrophages [7], adipocytes [8] keratinocytes [9], vascular endothelial cells [10] and skeletal muscle satellite cells [11], it is necessary to compare the cytopathic pattern produced by mycolactone on Schwann cells to that on other cells. Therefore, mouse fibroblast cell line L929 and macrophage cell line J774 were used for comparison studies. Synthetic mycolactone A/B [12] was used for the evaluation of mycolactone alone. In addition, the cytotoxic effect of synthetic mycolactone A/B remote diastereomer (stereocenter present outside a self-contained box) [12] was compared with that of synthetic mycolactone A/B.

## Materials and methods

### Eukaryotic cell culture

L929 mouse fibroblast cells (ATCC CCL-1) were purchased from the American Type Culture Collection and passaged in Dulbecco‘s Eagle's Minimum Essential Medium supplemented with 10% heat-inactivated horse serum at 37°C with 5% CO_2_. Mouse macrophage cells J774A.1 (ATCC TIB-67), C2C12 mouse myoblast (ATCC CRL-1772), Neuro-2a mouse neuroblast (ATCC CCL-131), sNF96.2 human Schwann cells (ATCC CRL-2884) were purchased from the American Type Culture Collection and passaged in Dulbecco‘s Modified Eagle’s Medium supplemented with 10% heat-inactivated fetal calf serum at 37°C with 5% CO_2_. HUVEC human endothelial cells (Lonza CC-2519) were purchased from Lonza and passaged in Endothelial Cell Growth Medium 2 Kit (Lonza C-22111) at 37°C with 5% CO_2_. SW10 mouse Schwann cells (ATCC CRL-2766) were purchased from the American Type Culture Collection and passaged in Dulbecco’s Modified Eagle’s Medium supplemented with 10% heat-inactivated fetal calf serum at 33°C with 5% CO_2_.

### Mycolactone

Synthetic mycolactone A/B and synthetic mycolactone A/B remote diastereomer were supplied by one of the coauthors (Yoshito Kishi), as ethanol-diluted solutions (1 mg/ml). The purity of synthetic mycolactone A/B and mycolactone A/B remote diastereomer was confirmed by 1H- and 13C-nuclear magnetic resonance and also by high performance liquid chromatography. The 0.20 mg/ml stock solution was prepared as follows: Firstly, 10.30 mg mycolactone A/B (the weight was determined by a Mettler ultra-micro balance) was dissolved in 10.3 ml ethanol to prepare a 1.0 mg/ml solution. Secondly, 2.0 ml of the above solution was diluted with 8.0 ml ethanol to prepare a 0.2 mg/ml solution. 0.50 ml each of the solution was transferred to a brown ampoule and sealed under argon. Ampoules containing the 0.20 mg/ml stock solution were kept in dark at -20°C. Thirdly, the concentration of the stock solution was further confirmed by the optical density at 362 nm (UV λ_max_ 362 nm (log ε 4.35)). The same procedure was used for preparation of the 0.20 mg/ml stock solution of mycolactone A/B remote diastereomer; in this series, 10.20 mg of synthetic mycolactone A/B remote diastereomer was used. They were diluted using culture medium to 30 μg/ml, 3 μg/ml, 300 ng/ml, 30 ng/ml, 3 ng/ml and 300 pg/ml. Ethanol similarly diluted with culture medium to a final ethanol concentration of 3 μg/ml was used as the negative control.

### Cytotoxic effect of synthetic mycolactone A/B on fibroblasts and macrophages

L929 fibroblasts and J774 macrophages were cultured in 24-well plates (2.0x10^4^ cells/well) for 24 hrs. Synthetic mycolactone A/B similarly diluted ten-fold from 30 μg/ml to 300 pg/ml was added and morphological evaluations were performed under a phase-contrast microscope (Nikon TMS) every 24 hrs for up to 60 hrs.

### Cytotoxic effect of synthetic mycolactone A/B on fibroblasts and Schwann cells

L929 fibroblasts and SW10 Schwann cells were cultured in 24-well plates (each well containing 2.0x10^4^ cells) for 24 hrs. Synthetic mycolactone A/B with a final concentration of 300 ng/ml, 30 ng/ml, or 3 ng/ml was added to the culture wells and incubated. The counting of dead cells after trypan blue staining and the TUNEL assay were performed at 24 hrs and 48 hrs as described below.

#### Assessment of cell viability using trypan blue staining

L929 fibroblasts and SW10 Schwann cells cultured for 24 hrs and 48 hrs following the addition of mycolactone A/B were detached by trypsin, collected in test tubes, and centrifuged at 1200 rpm for 5 min. After removing the supernatant, 1 ml of trypan blue staining solution was added. Total cell number and number of dead cells stained by trypan blue were counted on cell counting plates under a phase-contrast microscope.

#### TUNEL assay

L929 fibroblasts and SW10 Schwann cells were cultured on BD Falcon 2-well culture slides (2.0x10^4^ cells/well) for 24 hrs before the addition of mycolactone A/B. The cells were fixed with 4% paraformaldehyde-PBS for 20 min and washed with PBS at 24 hrs and 48 hrs. When many cells had detached and were floating, both floating and adherent cells detached by a cell scraper were collected into test tubes and centrifuged at 1200 rpm for 5 min. After removing the supernatant, the cells were fixed on silan-coated glass slides. The TUNEL assay was performed using the ApoTag Plus Peroxidase In Situ Apoptosis Detection Kit S7101 (Chemicon, U.S.A.) following the manufacturer's instructions. Mild hematoxylin staining was used instead of methyl green nuclear counterstaining. Photomicrographs were used to count total cell number and the number of TUNEL-positive cells.

### Effect of mycolactone on cellular morphology

SW10 mouse Schwann cells and L929 mouse fibroblast cells were cultured for 24 hrs in the same manner as described above. Synthetic mycolactone A/B diluted to a final concentration of 300 ng/ml, 30 ng/ml, or 3 ng/ml was added to the cells and incubated for 12, 24, 48 and 72 hrs. Photomicrographs were taken using the phase-contrast microscope.

### Detection of apoptosis by Western blot analysis

SW10 mouse Schwann cells, L929 mouse fibroblasts, J774A.1 mouse macrophage, C2C12 mouse myoblast, Neuro-2a mouse neuroblast, sNF96.2 human Schwann cells and HUVEC human endothelial cells were cultured for 24 hrs. Synthetic mycolactone A/B with a final concentration of 300 ng/ml, 30 ng/ml, or 3 ng/ml was then added and the cells were incubated further. Floating and adhered cells were collected at 12, 24 and 48 hrs time points. Ethanol similarly diluted with culture media to a final ethanol concentration of 300 ng/ml was used as the negative control. In addition, actinomycin-D (Sigma) diluted with culture medium for 24 hrs was used as the positive control. Following the bicinchoninic acid assay (BCA assay), Western blot analysis was performed using rabbit anti-cleaved caspase-3 (Cell Signaling #9661), rabbit anti-caspase-3 antibody (Cell Signaling #9662), mouse monoclonal anti-histone H2A.XS139ph (phospho Ser139) antibody (GENETEX, Inc. GT2311), and mouse monoclonal anti-α-tubulin (Sigma T-9026) as an internal control. Horseradish peroxidase (HRP)-labeled goat anti-mouse IgG (7076) and goat anti-rabbit IgG (7074), purchased from Cell Signaling, were used as secondary antibodies. Immunoreactive bands were visualized using a chemiluminescence reagent Immuno Star LD (Wako).

### Detection of apoptosis by fluorescence microscopy

Fibroblasts and Schwann cells were cultured in chamber slides for 24 hrs. Synthetic mycolactone A/B with a final concentration of 300 ng/ml, 30 ng/ml, or 3 ng/ml was added before further culturing for 12 and 24 hrs. Ethanol similarly diluted with culture media to a final ethanol concentration of 300 ng/ml was used as the negative control. In addition, actinomycin-D diluted with culture medium for 24 hrs was used as the positive control. Following fixation with paraformaldehyde and Triton-X treatment, the cultures were fluorescently stained with the following reagents: cleaved caspase-3 was stained fluorescent red (Rabbit anti-Cleaved Caspase-3 (1:1000)/Alexa Fluor 594 Goat Anti-Rabbit IgG), nuclear DNA was stained blue (Hoechst 33342), and intracellular actin was stained green (Alexa Fluor 488 Phalloidin). Cells were examined under a confocal laser scanning microscope (Olympus: FV10i-DOC Laser Scanning Microscope).

### Comparison of synthetic mycolactone A/B and its remote diastereomer

L929 fibroblasts and SW10 Schwann cells were cultured, and treated with the same concentration of mycolactone A/B and mycolactone A/B remote diastereomer. Trypan blue staining and the TUNEL assay were performed to count dead cells and evaluate apoptosis, respectively.

### Statistic analysis

The Mann-Whitney U-test was applied for the comparison of data obtained from the two groups.

## Results

### Cytotoxic effect of synthetic mycolactone A/B on fibroblast and macrophage cell lines

Synthetic mycolactone A/B exerted cytotoxicity against the tested cell lines. Both fibroblasts and macrophages exhibited detachment of most adhered cells 24 hrs after the addition of 30 μg/ml and 3 μg/ml of synthetic mycolactone A/B. Treatment with 300 ng/ml and 30 ng/ml of synthetic mycolactone A/B resulted in partial detachment, while no floating cells were found in the 3 ng/ml, 300 pg/ml and negative control cultures. At 60 hrs, the number of floating cells increased, but 3 ng/ml and 300 pg/ml cultures contained no floating cells.

### Cytotoxic effect of synthetic mycolactone A/B on fibroblast and Schwann cell lines

Fibroblasts underwent shrinkage and detachment 24 hrs after the addition of 300 ng/ml and 30 ng/ml of mycolactone. However, 3 ng/ml and 300 pg/ml produced no morphological changes.

**Fig 1:**
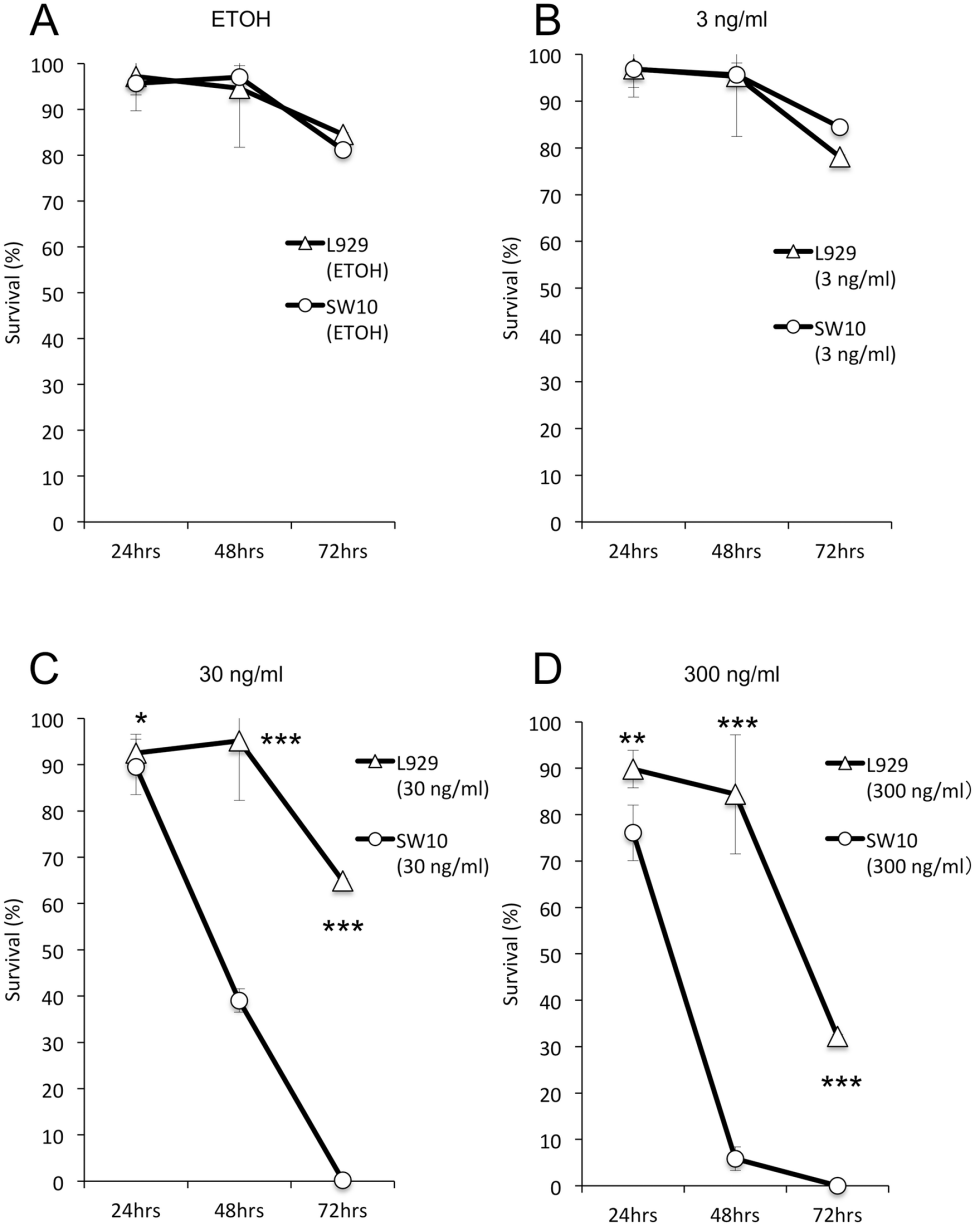
Assessment of cell viability. L929 fibroblasts and SW10 Schwann cells were cultured for 24, 48 and 72 hrs after the addition of mycolactone A/B. Morphological changes were observed under a phase-contrast microscope. Total cell number and cell viability in trypan blue-stained were counted on cell counting plates under a phase-contrast microscope. Originally both fibroblasts and Schwann cells were elongated and adhered, but after addition of synthetic mycolactone A/B they showed round shrinkage, detachment and floating. Viability counts demonstrated different sensitivities of the two cell lines. More than 80% of fibroblasts were adhered until 48 hrs (A, B), and approximately half the cells treated with 300 ng/ml and 30 ng/ml of mycolactone were adhered until 72 hrs (C, D). Conversely, Schwann cells showed a higher level of detachment starting at 24 hrs after the addition of 300 ng/ml and 30 ng/ml of mycolactone, and most exhibited detachment at 48 or 72 hrs (C, D). * *p* < 0.01; ** *p* < 0.002; *** *p* < 0.001, compared to corresponding L929 levels.

Schwann cells also displayed partial detachment 24 hrs after the addition of 300 ng/ml and 30 ng/ml of mycolactone. However, no morphological changes were detected in the 3 ng/ml and 300 pg/ml cultures. At 48 hrs, floating cells were observed in the culture containing 30 ng/ml of mycolactone. Cytotoxicity levels of mycolactone were compared between Schwann cells and fibroblasts. As shown in Fig 1, cell death as evaluated by trypan blue staining showed that Schwann cells are more sensitive to mycolactone than fibroblasts at the concentrations of 30 ng/ml (24 hrs, *p* < 0.01; 48 hrs, *p* < 0.001; 72 hrs, *p* < 0.001) and 300 ng/ml (24 hrs, *p* < 0.002; 48 hrs, *p* < 0.001; 72 hrs, *p* < 0.001).

**Fig 2:**
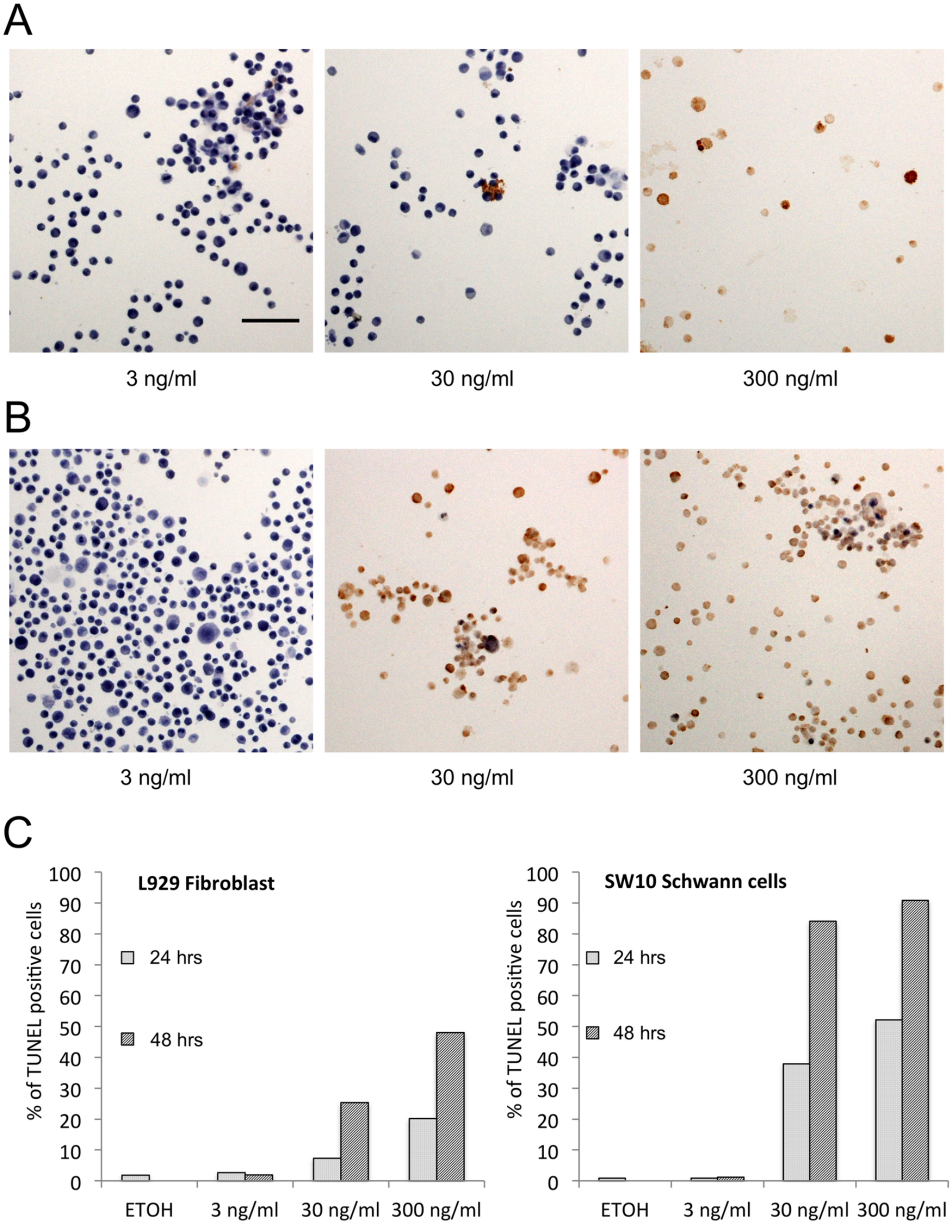
TUNEL assay in SW10 and L929 cells. L929 fibroblasts and SW10 Schwann cells were cultured on BD Falcon 2 well culture slides for 24 hrs (2.0x10^4^ cells/well) before synthetic mycolactone A/B was added. The cells were fixed with 4% paraformaldehyde-PBS and washed with PBS at 24 hrs and 48 hrs. The TUNEL assay and mild hematoxylin nuclear staining were performed. Total cell number and number of TUNEL-positive cells were counted using photomicrographs. At 24 hrs, 30 ng/ml of mycolactone induced less apoptosis (brown nuclear staining) in fibroblasts (A) than in Schwann cells (B). Three ng/ml of mycolactone did not show significant TUNEL reaction, while 300 ng/ml produced a strong TUNEL reaction in both fibroblasts and Schwann cells. In the quantitative analysis (C), fibroblasts in 3 ng/ml of mycolactone showed no apoptosis at 24 and 48 hrs, but 30 and 300 ng/ml of mycolactone induced apoptosis in a concentration-dependent and time-dependent manner. Schwann cells also showed no apoptosis at 24 and 48 hrs with 3 ng/ml of mycolactone; however, 30 and 300 ng/ml of mycolactone induced more apoptosis in Schwann cells (91% at 48 hrs, 300 ng/mg) than in fibroblasts (48% at 48 hrs, 300 ng/ml). Bar = 100 μm.

Apoptosis induced by mycolactone was evaluated for the Schwann cells and fibroblasts. As shown in Fig 2, the TUNEL assay also showed that Schwann cells are more sensitive to mycolactone than fibroblasts.

### Change of cellular morphology by mycolactone

**Fig 3:**
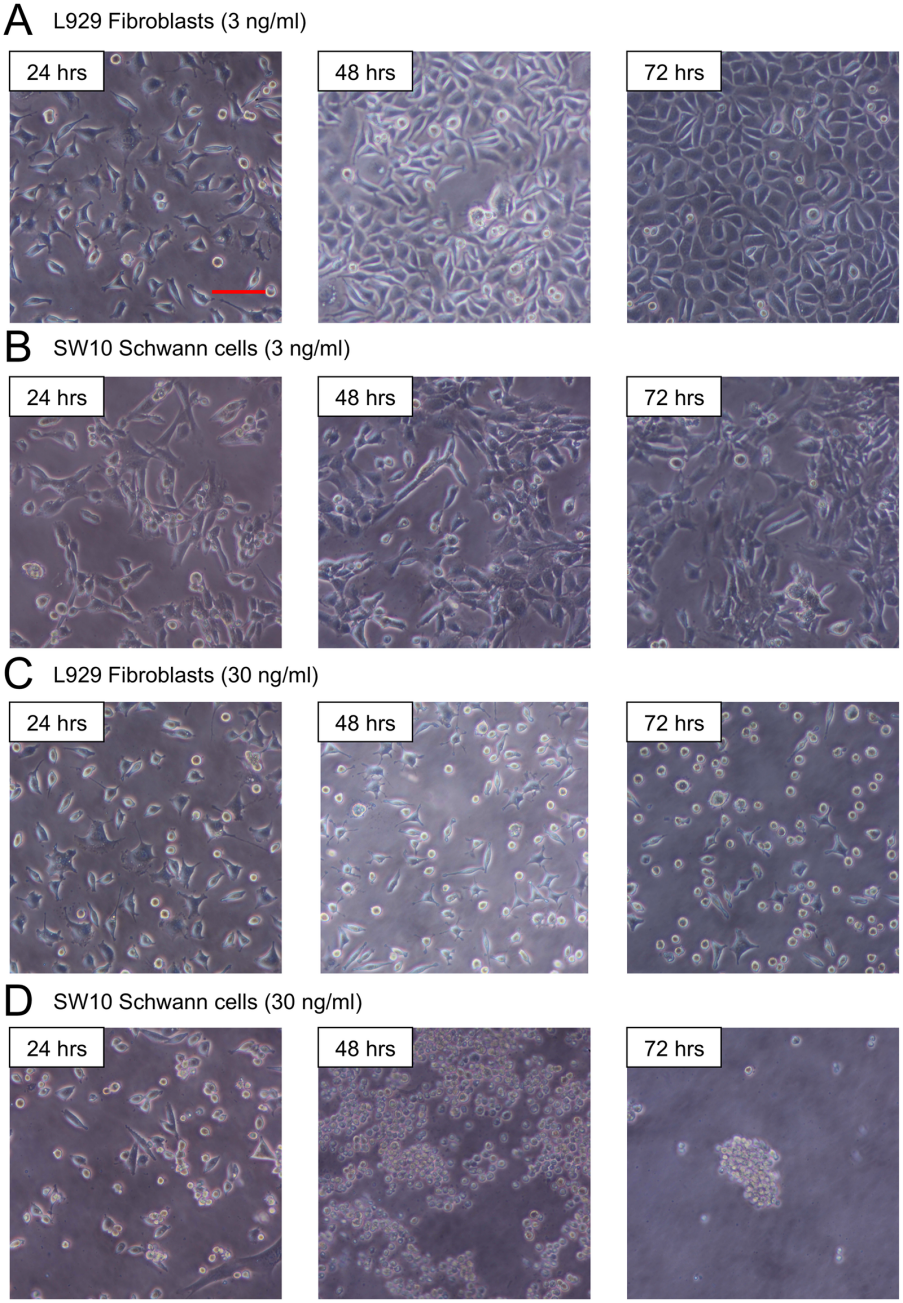
Effect of mycolactone on cellular morphology. SW10 mouse Schwann cells and L929 mouse fibroblast cells were cultured for 24 hrs. Synthetic mycolactone A/B diluted to a final concentration of 3 ng/ml, 30 ng/ml, or 300 ng/ml was added to a cell culture and incubated for 12, 24, 48 and 72 hrs. Photomicrographs were taken by a phase-contrast microscope. Cells treated with 3 ng/ml of mycolactone showed no floating cells at 24, 48, or 72 hrs (A, B). Fibroblasts showed no changes until 48 hrs, but partial detachment began at 72 hrs with 30 ng/ml of mycolactone A/B (C). Schwann cells showed round shrinkage and floating at 24 hrs with 30 ng/ml of mycolactone A/B. Some of the cells remained adherent at 48 hrs, but all cells were detached at 72 hrs (D). Bar = 100 μm.

As shown in Fig 3, control cells and cells treated with 3 ng/ml of mycolactone contained no floating cells at 24, 48, or 72 hrs. Fibroblasts exhibited no changes until 48 hrs, but partial detachment began at 72 hrs with 30 ng/ml of mycolactone A/B. In contrast, Schwann cells displayed round shrinkage and floating at 24 hrs with 30 ng/ml of mycolactone A/B. Some adherent cells remained at 48 hrs, but all had detached at 72 hrs.

### Detection of apoptosis by Western blot analysis

**Fig 4:**
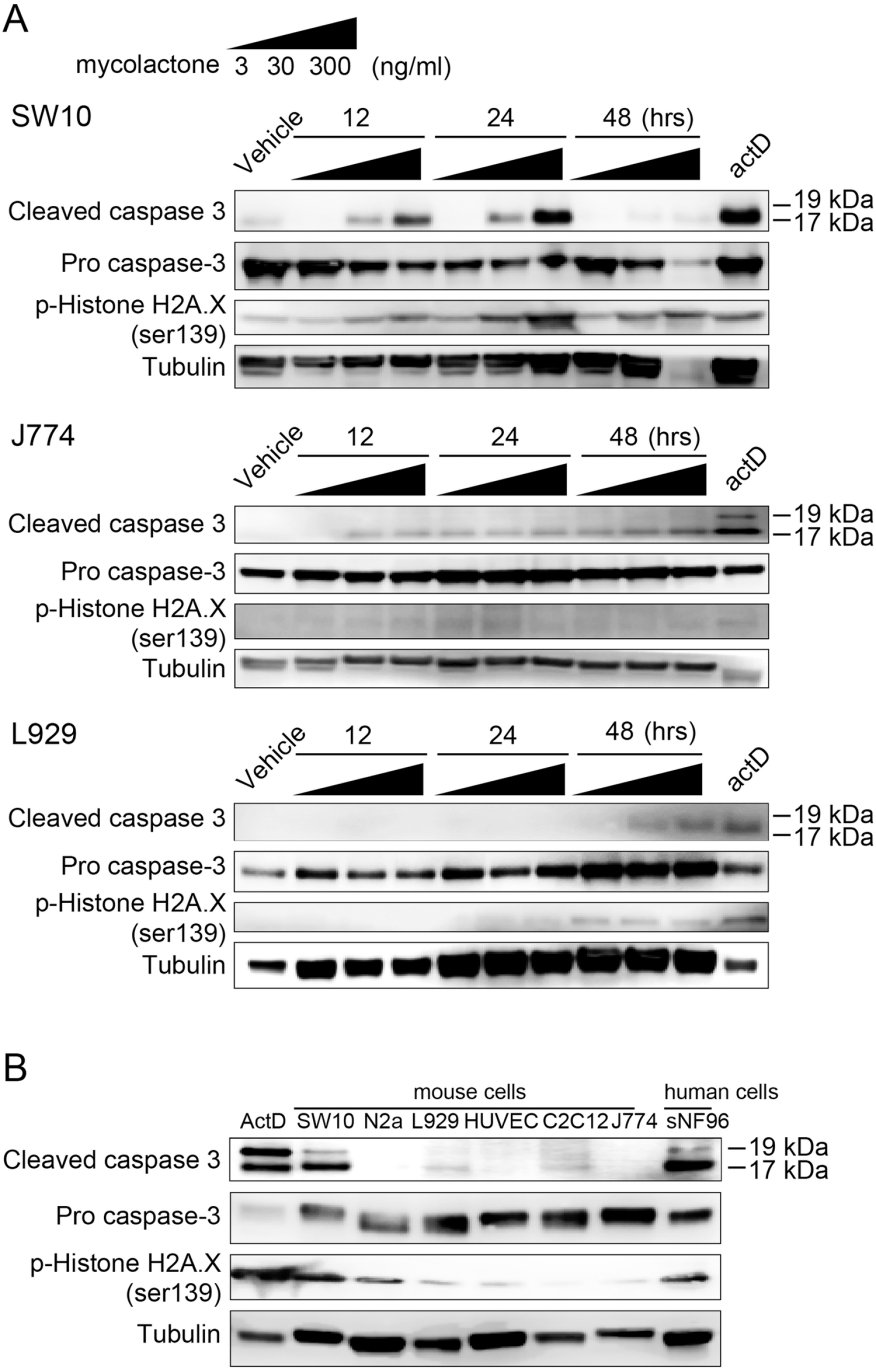
Detection of apoptosis by Western blot analysis. SW10 mouse Schwann cells, L929 mouse fibroblasts, J774A.1 mouse macrophage, C2C12 mouse myoblast, Neuro-2a mouse neuroblast, sNF96.2 human Schwann cells and HUVEC human endothelial cells were cultured for 24 hrs. Synthetic mycolactone A/B (3 ng/ml, 30 ng/ml, or 300 ng/ml) was added before further incubation for 12, 24 and 48 hrs. The BCA assay and Western blotting of floating and adhered cells was performed using rabbit anti-cleaved caspase-3, rabbit anti-caspase-3 antibody, mouse monoclonal histone H2A.XS139ph (phospho Ser139) antibody, and mouse monoclonal anti-α-tubulin (internal control). As a positive control, Actinomycin-D was used. **(A)** In SW10 Schwann cells cleaved caspase 3 was definitely positive at 12 and 24 hrs with 30 and 300 ng/ml. Phosphorylated histone H2A.X showed similar induction pattern, but lasted until 48 hrs. In J774 macrophages, mycolactone slightly induced cleaved caspase 3, but it did not induce p-histone H2A.X. Also, mycolactone only slightly induced cleaved caspase 3 and p-histone H2A.X at 48 hrs in the fibroblasts. **(B)** Cellular response to mycolactone concentration 30 ng/ml at 24 hrs was compared. Mouse Schwann cells (SW10) and human Schwann cells (sNF96.2) showed strong induction of both cleaved caspase 3 and p-histone H2A.X by mycolactone. Mouse neuroblasts showed induction of p-histone H2A.X. However, fibroblasts (L929), myoblasts (C2C12), endothelial cells (HUVEC) and macrophages (J774) did not show clear induction.

As shown in Fig 4A, SW10 Schwann cells showed induction of cleaved caspase 3 and phosphorylated histone H2A.X at 12 and 24 hrs with 30 and 300 ng/ml. At 48 hrs, cleaved caspase 3 became negative, but p-histone H2A.X was expressed. In J774 macrophages, mycolactone slightly induced cleaved caspase 3, but it did not induce p-histone H2A.X. Mycolactone slightly induced cleaved caspase 3 and p-histone H2A.X at 48 hrs in the fibroblasts. Fig 4B shows comparison of seven cell lines at 24 hrs with 30 ng/ml of mycolactone. Mouse Schwann cells (SW10) and human Schwann cells (sNF96.2) showed strong induction of both cleaved caspase 3 and p-histone H2A.X by mycolactone. Mouse neuroblasts also showed induction of p-histone H2A.X. However, fibroblasts (L929), endothelial cells (HUVEC), myoblasts (C2C12) and macrophages (J774) did not show clear induction.

### Detection of apoptosis by fluorescence microscopy

**Fig 5:**
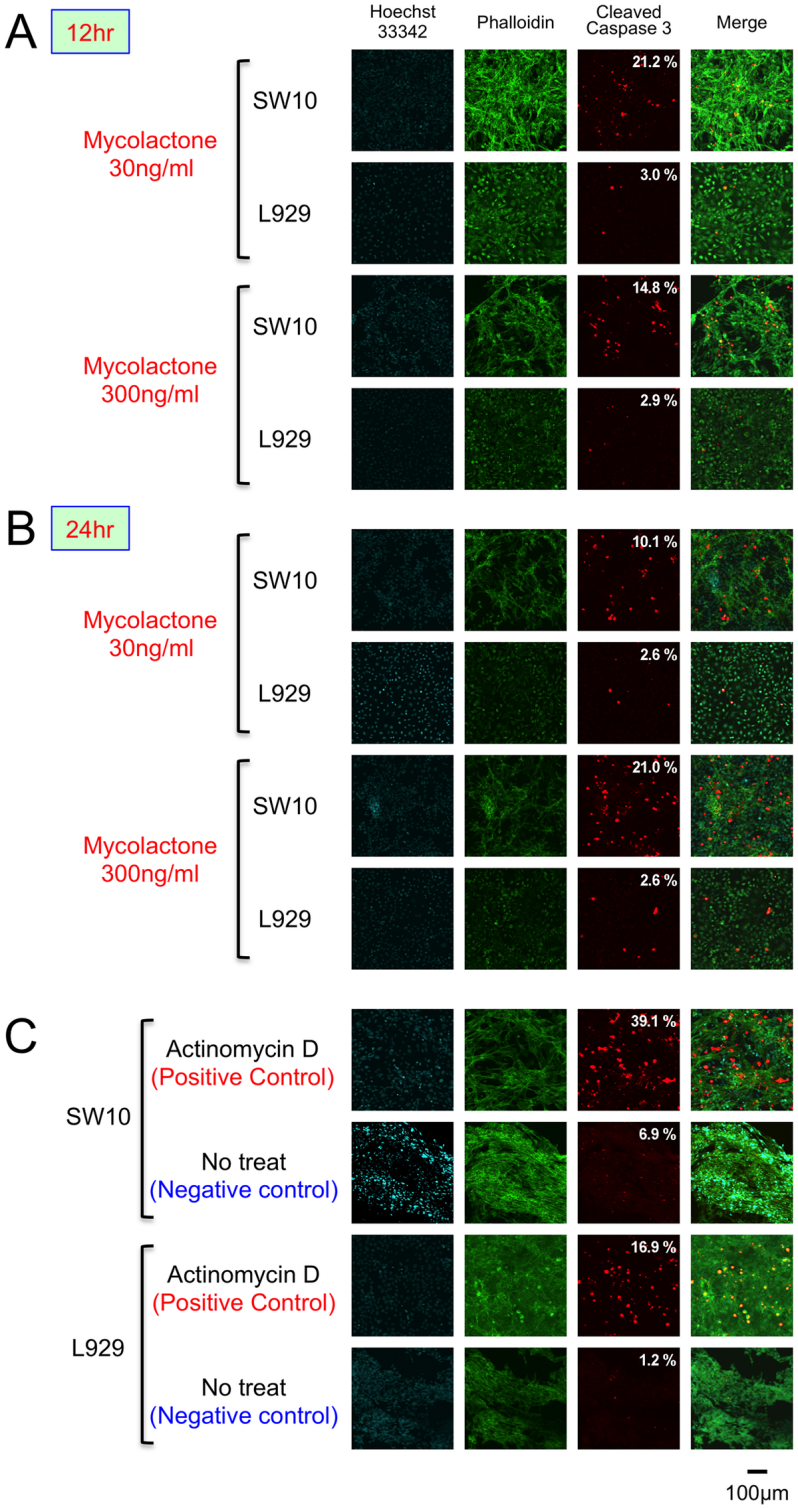
Detection of apoptosis by fluorescence microscopy. Fibroblasts and Schwann cells were cultured in chamber slides for 24 hrs. Synthetic mycolactone A/B with a final concentration of 3 ng/ml, 30 ng/ml, or 300ng/ml was added and further cultured for 12 and 24 hrs. Fixed cells were stained with fluorescent reagents. Red: cleaved caspase-3 (Rabbit anti-Cleaved Caspase-3 (1:1000)/Alexa Fluor 594 Goat Anti-Rabbit IgG); Blue: nuclear DNA (Hoechst 33342); and Green: intracellular actin (Alexa Fluor 488 Phalloidin). Cells were examined under a confocal laser scanning microscope (Olympus: FV10i-DOC Laser Scanning Microscope). As a positive control, actinomycin-D was added to the culture. Expression of cleaved caspase-3 was compared at 12 and 24 hrs after administration of mycolactone. Positive cell rate (number of cleaved caspase 3 positive cells/number of Hoechst 33342 positive cells, %) was calculated. (A and B) In the four conditions (12 and 24 hrs, 30 and 300 ng/ml mycolactone), the expression of cleaved caspase 3 was observed in the cytoplasm of SW10 Schwann cells (10–21%) and in some of L929 fibroblasts (2–3%). (C) Actinomycin-D showed the expression of cleaved caspase 3 to SW10 and L929 cells.

Expression of cleaved caspase-3 was compared at 12 and 24 hrs after the administration of mycolactone. In the four conditions (12 and 24 hrs, 30 and 300 ng/ml mycolactone), the expression of cleaved caspase 3 was observed in the cytoplasm of SW10 Schwann cells (10–21%) and in some of fibroblasts (2–3%) (Fig 5).

### Comparison of synthetic mycolactone A/B and its remote diastereomer

**Fig 6:**
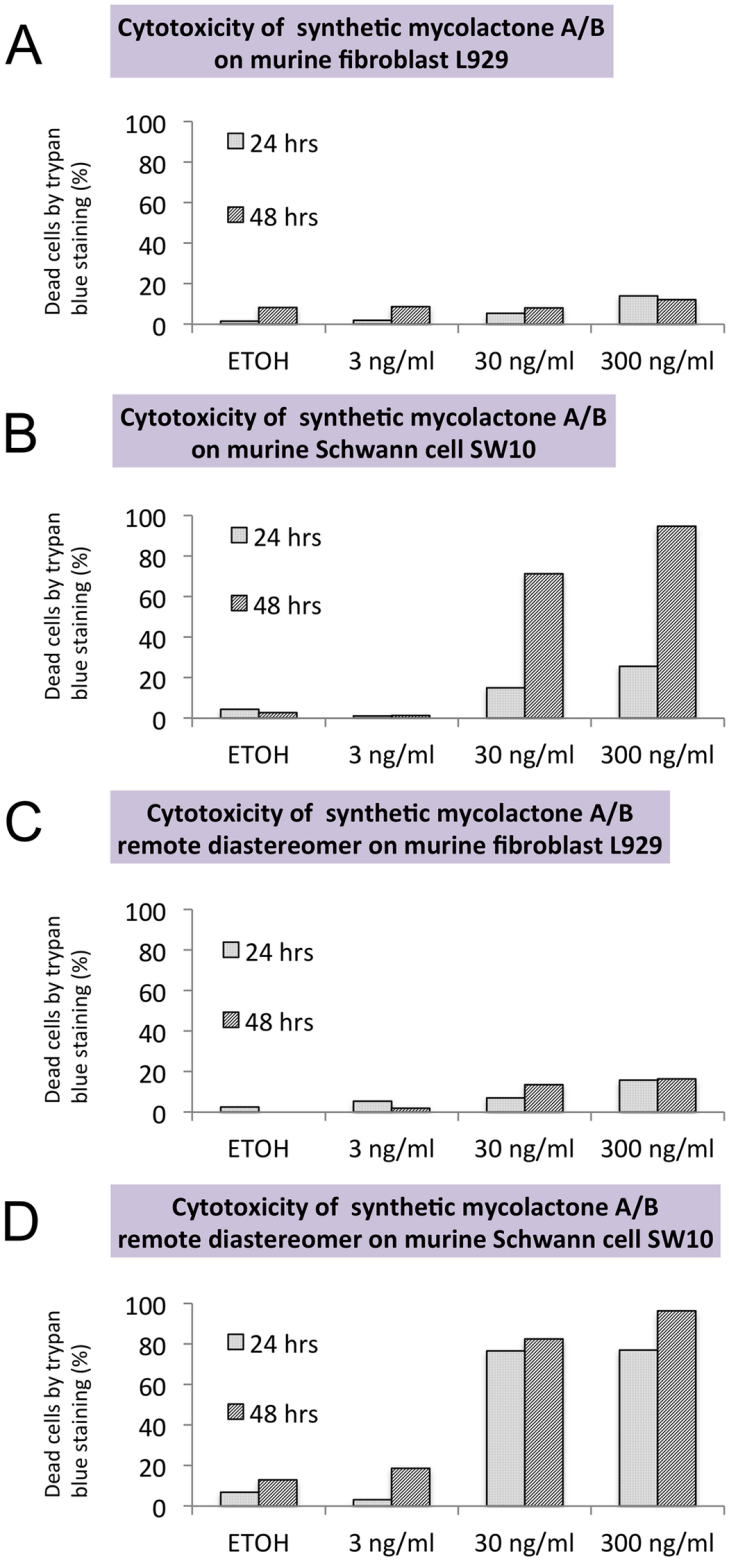
Comparison of synthetic mycolactone A/B and its remote diastereomer. L929 fibroblasts and SW10 Schwann cells were cultured and treated with the same concentration of mycolactone A/B or mycolactone A/B remote diastereomer. Trypan blue staining and the TUNEL assay were performed. Synthetic mycolactone A/B (A, B) and its remote diastereomer (C, D) exerted identical cytotoxicity in both fibroblasts and Schwann cells at the same concentration.

As shown in Fig 6, synthetic mycolactone A/B and its remote diastereomer exerted identical cytotoxicity to both fibroblasts and Schwann cells at the same concentration.

## Discussion

Patients with Buruli ulcers are thought to have no pain unless secondary bacterial infection occur [1]. However, Alferink *et al*. [2] showed the presence of severe pain during wound care in about 30% of patients. Addison *et al*. [13] also reported that 54.8% of patients experienced wound pain associated with wound dressing at a primary health care center, where secondary bacterial infection was 9%. These studies suggest that Buruli ulcer lesions are initially painless, but the patients experience pain after chemotherapy, probably due to nerve regeneration. Antibiotic therapy decreases mycolactone concentration in Buruli ulcer lesions [14]. In our previous study with a single injection of mycolactone to mouse footpads [4], we had already demonstrated thin myelins (remyelination) 28 days after the injection and normal myelins (completion of remyelination) on day 42, indicating that mycolactone-induced nerve damages are reversible. These findings lead us to further study cytopathic mechanism of mycolactone to Schwann cells.

To determine whether synthetic and purified mycolactone have similar biological activities and the optimal mycolactone concentration for the cytotoxic studies, various amounts of mycolactone were added to L929 fibroblasts and J774 macrophages and cultured for 24, 48 and 72 hrs. At 24 hrs, no detachment was observed with mycolactone concentrations of 3 ng/ml and 300 pg/ml, but many floating cells were found following treatment with concentrations of 30 μg/ml, 3 μg/ml, 300 ng/ml and 30 ng/ml. These results were compatible with the study of George *et al*. [4] using mycolactone purified from cultured *M*. *ulcerans*. The biological activity of synthetic mycolactone in cultured cells was confirmed in this study. The threshold concentration of cytotoxicity was between 30 ng/ml and 3 ng/ml. Sarfo et al. [14] measured the concentration of mycolactone in 80 untreated human Buruli ulcer lesions by mass spectrometry, and the median (range) concentration was 26 ng/ml (0–1970), which also supports the adequacy of our present study.

Trypan blue vital staining reflects cell membrane damage, which might be caused either by the necrotic pathway or late-stage apoptotic process. The counting of trypan blue-positive cells showed that a much higher percentage of SW10 Schwann cells (76.7%) than L929 fibroblasts (7.1%) were dead 24 hrs after exposure to 30 ng/ml of mycolactone. No significant increase of trypan blue-positive cells was observed at 48 hrs.

The TUNEL reaction stains cellular nuclei undergoing the apoptotic process, but not those of the necrotic pathway. In the present study, TUNEL-positive cells were much more frequent in SW10 Schwann cells than in L929 fibroblasts 24 hrs following exposure to 30 ng/ml of mycolactone. The amounts linearly increased to 84.1% (SW10) and 25.4% (L929) at 48 hrs. Chronological differences between the cell death study and apoptosis study may be reflective of the cytotoxic mechanism of mycolactone, but further study is required.

As well as caspase-7, Caspase-3 is a key protease involved in cellular apoptotic processes [15]. That mycolactone induced cleaved caspase-3, a late apoptosis marker, as well as phospho-histone H2A.X, a marker for early apoptosis, in Schwann cells but not in fibroblasts or macrophages was confirmed by Western-blotting and immunocytochemistry in the present study (Fig 4A). Moreover, in the comparison of various cell lines (Fig 4B), mouse and human Schwann cells showed strong induction of both cleaved caspase 3 and p-histone H2A.X by mycolactone. Mouse neuroblasts also showed induction of p-histone H2A.X. In contrast, clear induction was not observed in fibroblasts, myoblasts, endothelial cells or macrophages. These overall results suggest that Schwann cells and neurons are more sensitive to mycolactone than other cell types.

Given the previous study demonstrating that mycolactone diffuses passively across the cell membrane [16], it is conceivable that susceptibility to mycolactone could be defined by several factors (e.g., distribution, expression level, or affinity) associated with cellular target molecules of mycolactone rather than particular membrane receptors. In this context, mycolactone is known to directly bind to WASP/NWASP, resulting in cytoskeletal rearrangements and detachment of target cells [17]. Considering the physiological importance of WASP in the development of Schwann cells [18], it is possible that cellular susceptibility to mycolactone may be affected by cell type specific WASP/NWASP expression and distribution, or their affinity to mycolactone. Clearly, further studies will be needed to clarify which factors define cellular susceptibility to mycolactone.

Recently, Marion *et al*. [19] demonstrated that mycolactone induces hypoesthesia by eliciting signaling through type 2 angiotensin II receptors, leading to potassium-dependent hyperpolarization of neurons. In their study, murine sciatic nerves innervating the infected footpads did not show morphological changes. In our previous studies [3] [4], murine nerve bundles at the inoculation sites showed degeneration, but sciatic nerves were not examined as they are distant from the lesion and pathology was not expected in our animal model. In our experimental model, we have demonstrated apoptosis at 12 hrs (Western blot) by mycolactone at a concentration similar to that found in human Buruli ulcer lesions (30ng/ml). On the other hand, Marion et al. applied 350ng/ml of mycolactone to primary hippocampal neuronal culture and demonstrated voltage change but no cell death within 20 min. As the temporal axes of two studies are different, direct comparison is impossible, but approximately 30 to 300 ng/ml of mycolactone seem to have both short-term effect and long-term effect to cultured cells.

Guenin-Macé *et al*. [17] showed that mycolactone modifies actin assembly and distribution in the cytoplasm by hijacking the Wiskott-Aldrich syndrome protein (WASP) family, resulting in defective cell adhesion and directional migration of epithelial cells. Detachment of cultured cells in our study may be evoked by this WASP-mediated process [17]. They also found that a distinct truncated version of mycolactone minimal structure inhibits the inflammatory cytokine responses of human primary cells at noncytotoxic doses in vitro [20]. Such studies could reveal new therapeutic uses for modified mycolactone substances. Recent studies revealed that mycolactone inhibits the production of inflammatory mediator by blocking the translocation into endoplasmic reticulum [21]. This immunosuppressive process is evoked by selective blockage of Sec-61 translocon by mycolactone [22]. Lack of inflammatory cells in the Buruli ulcer lesion is reasonably explained by these studies.

In summary, we demonstrated the cytotoxicity of synthetic mycolactone A/B and its remote diastereomer in mouse Schwann cells as well as fibroblasts and macrophages. A quantitative study showed that Schwann cells are relatively more sensitive than fibroblasts to mycolactone 24 and 48 hrs after exposure to mycolactone A/B diastereomer. The painless nature of Buruli ulcer may be caused by the cytotoxicity of mycolactone A/B in cultured Schwann cells.
